# Supplementary material for: The potential role of religiosity, psychological immunity, gender, and age group in predicting the psychological well-being of diabetic patients in Saudi Arabia within the Bayesian framework
Source: PLoS One. 2024 Aug 23;19(8):e0308454. doi: 10.1371/journal.pone.0308454 (PMC11343467; doi:10.1371/journal.pone.0308454)
Supplement: Attachment — Submitted filename: Response to reviewers.docx [file pone.0308454.s001.docx]

Dear Editor

Greetings

Thank you for your great efforts and the reviewers, whose comments contributed to enhancing our manuscript. All comments have been taken into account, and We have edited all of the reviewer comments, we hope that the revised manuscript meets your acceptance.

Best regards

1. Formatting Issues:

Ensure proper punctuation after headings, such as a colon.
Capitalize the first letter of scale names in the method section.
Ensure that Table 4 appears on a single page.

Reply:

Thank you for your important comment, we edited all of these and highlighted them with yellow.

2. Literature Review:

Enhance the literature review by incorporating additional research to provide a more comprehensive analysis. Strengthen the argumentative and analytical aspects of the literature review to improve coherence and logical flow.

Reply:

Thank you for your important comment, we edited and highlighted it with yellow.

Introductory Paragraph:

Revise the introductory paragraph to provide a clearer overview of the research topic, objectives, and methodology.

Reply:

Thank you for your important comment, we edited and highlighted it with yellow.

Bayesian Model Integration:

Clarify the integration of the Bayesian model into the research methodology, particularly in relation to measuring religiosity and immunity in the Kingdom of Saudi Arabia, despite including non-Saudi populations in the sample.

Reply:

Thank you for your important comment, we edited this in the data analysis section and highlighted it with yellow.

Consistency in Draft:

Correct the consistent error in writing t-test names by ensuring the first letter is lowercase and italicized throughout the draft.

Reply:

Thank you for your important comment, we corrected it in the manuscripts and highlighted all of them in yellow.

Check for typographical errors, such as missing letters, in figures and throughout the text.
In-text Citation Consistency:

Reply:

Thank you for your important comment, we corrected all and highlighted all of them in yellow.

Standardize the citation format throughout the document to ensure consistency. Decide on a specific format, such as APA or MLA, and apply it uniformly.

**Reply:**

Thank you for your important comment, we checked it all and highlighted it with yellow.

Discussion Section:

Expand and deepen the discussion section to provide a more thorough analysis of the research findings and their implications.

**Reply:**

Thank you for your important comment, we edited and highlighted it with yellow.

In summary, the document requires revisions to address formatting issues, enhance the literature review, clarify the integration of the Bayesian model, maintain consistency throughout the draft, and strengthen the discussion section.

**Reply:**

We are grateful for your valuable feedback that has contributed to improving the quality of our manuscript. All comments have been taken into account, and we edited the comments and hope they meet with your approval.

Reply to the comments of the reviewer 2:

Overall the research paper impressively investigates into a topic but I would like to share few recommendations.
1. Paper has included many theories relevant to the study variables but I would suggest to summarize these theories and also specify which one theory you are focusing on while working on your research.

**Reply:**

Thank you for your important comment, we summarized it and wrote that we developed the PWB scale according to the PWB Ryff model, and highlighted it with yellow.

2. The paper's quality is elevated by the careful attention given to its methodology; however, the details of sampling technique used in the research will be a valuable addition. Also the details of how the assessment measure were developed by the researchers of the study should also be included.

**Reply:**

Thank you for your important comment, we edited and highlighted it with yellow.

3. Rationale and objectives of the study are missing. It must be included and the reason for using Bayesian framework should also be highlighted specifying its advantages and disadvantages.

**Reply:**

Thank you for your important comment, we added reasons for using the Bayesian framework and highlighted it with yellow.

4. The tile looks fine but it is just a recommendation after reading the whole paper that it should be rephrased and the role of gender and age should be incorporated in the title which are related to the main objectives of the study.

**Reply:**

Thank you for your important comment, we edited the study title according to this recommendation and highlighted it with yellow.

5. Future recommendations should also be added in the paper.

**Reply:**

Thank you for your important comment, we wrote it in the (limitations and future directions) section and highlighted it with yellow.

**Dear reviewer,**

We are grateful for your valuable feedback that has contributed to improving the quality of our manuscript. All comments have been taken into account, and we edited the comments and hope they meet with your approval.

Journal Requirements:

1. When submitting your revision, we need you to address these additional requirements.

Please ensure that your manuscript meets PLOS ONE's style requirements, including those for file naming. The PLOS ONE style templates can be found at 
<https://journals.plos.org/plosone/s/file?id=wjVg/PLOSOne_formatting_sample_main_body.pdf> and 
<https://journals.plos.org/plosone/s/file?id=ba62/PLOSOne_formatting_sample_title_authors_affiliations.pdf>.

**Reply:**

Thank you for your important comment, we edited the manuscript according to PLOS ONE's style requirements. and highlighted it with yellow.

2. e note that the grant information you provided in the ‘Funding Information’ and ‘Financial Disclosure’ sections do not match.

When you resubmit, please ensure that you provide the correct grant numbers for the awards you received for your study in the ‘Funding Information’ section.

**Reply:**

Thank you for your important comment, we edited and highlighted it with yellow.

This work was supported by Princess Nourah bint Abdulrahman University Project number (PNURSP2023R380)**.**

3. Thank you for stating the following in the Acknowledgments Section of your manuscript: 
[Princess Nourah bint Abdulrahman University Researchers Supporting Project number (PNURSP2023R380), Princess Nourah bint Abdulrahman University, Riyadh, Saudi Arabia. ]
We note that you have provided funding information that is not currently declared in your Funding Statement. However, funding information should not appear in the Acknowledgments section or other areas of your manuscript. We will only publish funding information present in the Funding Statement section of the online submission form. 
Please remove any funding-related text from the manuscript and let us know how you would like to update your Funding Statement. Currently, your Funding Statement reads as follows: 
 [The author(s) received no specific funding for this work.]

Please include your amended statements within your cover letter; we will change the online submission form on your behalf.

**Reply:**

Thank you for your important comment, please, our university demanded the following format of the Acknowledgment Section, necessary remain as it is :

Princess Nourah bint Abdulrahman University Researchers Supporting Project number (PNURSP2023R380), Princess Nourah bint Abdulrahman University, Riyadh, Saudi Arabia.

4. Please review your reference list to ensure that it is complete and correct. If you have cited papers that have been retracted, please include the rationale for doing so in the manuscript text, or remove these references and replace them with relevant current references. Any changes to the reference list should be mentioned in the rebuttal letter that accompanies your revised manuscript. If you need to cite a retracted article, indicate the article’s retracted status in the References list and also include a citation and full reference for the retraction notice.

**Reply:**

Thank you for your important comment, we reviewed the references and edited and highlighted them with yellow.
